# Trait-Based Optimization of Plant Density in Drip-Fertigated Wheat: Yield Formation and Nitrogen–Radiation–Water Use Efficiency Responses of Varieties Contrasting in Individual Spike Productivity

**DOI:** 10.3390/plants15081167

**Published:** 2026-04-09

**Authors:** Xiaoyan Zhou, Mei Qian, Faming Wang, Dapeng Gao, Guitao Zhao, Shiwei Wang, Depeng Wang, Xiaojun Hu

**Affiliations:** 1 College of Life Science, Linyi University, Linyi 276000, China; zhouxiaoyan@lyu.edu.cn (X.Z.); m15964386367@163.com (M.Q.); w15163901393@163.com (F.W.); gaodapeng@lyu.edu.cn (D.G.); wangdepeng@lyu.edu.cn (D.W.); 2 Linyi Agricultural Technology Extension Center, Linyi 276001, China; zhgt2044@126.com (G.Z.); shiweiwang1978@163.com (S.W.)

**Keywords:** plant density optimization, winter wheat, individual spike productivity, drip fertigation, resource use efficiency

## Abstract

Optimizing plant density is critical for improving wheat yield and resource-use efficiency, but whether a single density recommendation applies to varieties differing in individual spike productivity under drip fertigation remains unclear. A two-year field experiment (2023–2024 and 2024–2025) was conducted with two winter wheat varieties contrasting in spike type: a multi-spike type (Jimai23, MS) and a large-spike type (Jimai24, LS). Four target densities (200, 300, 400, and 500 plants m^−2^) were evaluated under drip fertigation to quantify yield formation, dry matter production, radiation interception and use, N uptake and nutritional status, and water use. Grain yield responses to density differed markedly between varieties. MS showed an increase–plateau–decline pattern, with the highest yields at 300–400 plants m^−2^ (10.13–10.97 t ha^−1^), whereas LS increased to 400 plants m^−2^ and remained relatively stable at 500 plants m^−2^ (9.97–10.55 t ha^−1^). Increasing density increased spike number, LAI, intercepted solar radiation (ISR), and soil water consumption but decreased grains per spike, grain weight, and yield per spike in both varieties. Yield variation was more strongly associated with post-anthesis dry matter production than with grain number. Although MS intercepted more radiation, its radiation use efficiency (RUE), post-anthesis N uptake, N nutrition index (NNI), harvest index, agronomic N-use efficiency, fertilizer N recovery efficiency, and water use efficiency (WUE) declined sharply at high density. In contrast, LS maintained relatively stable RUE, higher NNI, stronger N uptake, and higher WUE at medium-to-high densities. These results demonstrate that optimal density under drip fertigation is variety-dependent and should be determined using a trait-based framework integrating nitrogen–radiation–water use efficiency.

## 1. Introduction

Achieving high and stable wheat (*Triticum aestivum* L.) yield while improving resource-use efficiency remains a central challenge in intensive cropping systems [1]. Plant density is one of the most influential and practical agronomic factors controlling wheat yield formation because it regulates population establishment, tillering dynamics, final spike number, canopy structure, and inter-plant competition [2]. Across a wide range of environments, wheat grain yield often shows a quadratic or plateau-type response to increasing density, but the agronomic optimum is not fixed and may vary with growing conditions and genetic background [3,4,5,6,7].

It has been shown that the yield response to plant density is fundamentally determined by the coordination among yield components and by the balance between population size and individual plant (or spike) productivity [8,9,10]. Increasing density generally increases spike number per unit area but often reduces grains per spike and/or grain weight because intensified inter-plant competition alters assimilate supply and sink realization during stem elongation and grain filling [11,12]. As a result, the net yield response to density is often characterized by component compensation rather than a simple linear increase, and excessive density may fail to increase yield or even cause yield penalties under some conditions [3,13]. Importantly, varieties differing in tillering ability and individual spike productivity may exhibit markedly different magnitudes and mechanisms of density response because they rely on different pathways to build sink size (e.g., spike number vs. grains per spike) and differ in their capacity to compensate at low or high density [3,14]. From a physiological perspective, these density effects should be interpreted within a source–sink framework, as yield formation depends on the interaction between sink components (spike number, grains per spike, grain size) and source traits related to canopy photosynthetic activity and assimilate supply during grain filling [15,16]. Therefore, clarifying how density reshapes yield-component coordination and source–sink balance in varieties contrasting in individual spike productivity is essential for developing trait-based density recommendations.

The physiological basis of such trait-dependent density responses is increasingly understood but remains complex. Final grain yield is jointly determined by dry matter accumulation and the efficiency of partitioning assimilates to grain (harvest index) [17,18,19]. In particular, post-anthesis dry matter production is often a key determinant of yield differences among management treatments because it directly reflects source activity during grain filling [20,21]. Biomass production can be further interpreted through the classic framework of intercepted solar radiation (ISR) and radiation use efficiency (RUE) [22,23]. Although higher density usually enhances canopy size (i.e., leaf area index) [24] for improved ISR, it may also intensify canopy shading and reduce within-canopy light distribution, leading to declines in RUE when canopy crowding becomes excessive [5,6,25]. However, some studies have shown that varieties of different types may differ substantially in both ISR and RUE [26,27]. This also suggests that, from the perspective of radiation interception and utilization mechanisms, different variety types may exhibit markedly different responses to plant density.

The development of crop sink size, canopy size and structure, as well as canopy RUE, is directly influenced by nitrogen uptake and crop N nutritional status, because N availability regulates leaf area expansion, canopy light interception, and the allocation of N within the canopy for photosynthetic function [28,29,30,31,32]. In addition, N uptake and crop N nutritional status are closely coupled with density regulation, canopy function, and yield formation. Higher density generally increases population N demand and may increase total N uptake, but it can also intensify N competition and reduce the N supply available per spike, thereby constraining sink establishment and grain filling. The nitrogen nutrition index (NNI), based on the critical N dilution framework, provides a robust in-season indicator of crop N status and is particularly useful for comparing N nutritional status across varieties and plant densities with contrasting biomass levels [33,34,35,36]. Meanwhile, density regulation also affects soil water consumption and water use efficiency (WUE), and the final outcome depends on whether the increase in water consumption is accompanied by a sufficient gain in grain yield [37,38,39]. Therefore, the effects of variety × density interaction on wheat productivity should be interpreted through an integrated nitrogen–radiation–water resource-use framework.

Although the effects of variety traits, density, and their interactions on wheat yield formation and related physiological processes have been extensively studied under conventional irrigation and fertilization management, corresponding evidence remains limited under drip fertigation (DF), which has been increasingly adopted in recent years [40,41,42,43,44,45]. This is especially true for the interaction between variety traits (particularly individual spike productivity) and density under DF conditions. This knowledge gap is agronomically important because a non-differentiated or inappropriate density choice under DF may offset the potential benefits of improved water–N supply synchronization and lead to substantial yield penalties and resource-use inefficiency. Therefore, the key question is whether the more stable water–N supply under DF weakens the dependence of optimum density on variety type or whether variety-specific density responses remain pronounced. We hypothesized that under drip fertigation, despite a more stable water–N supply, the optimal plant density remains strongly dependent on variety type (particularly individual spike productivity traits), and thus a single uniform density recommendation is not appropriate.

To test this hypothesis, we compared two winter wheat varieties contrasting in individual spike productivity (a multi-spike type and a large-spike type) across a density gradient under DF. We aimed to (i) quantify variety-specific yield responses to density, (ii) identify the underlying mechanisms in yield-component coordination and source–sink regulation, and (iii) clarify how coordinated nitrogen–radiation–water use efficiency determines density-dependent yield formation in varieties differing in individual spike productivity.

## 2. Results

### 2.1. Yield Performance and Yield-Related Agronomic Traits

**Figure 1:**
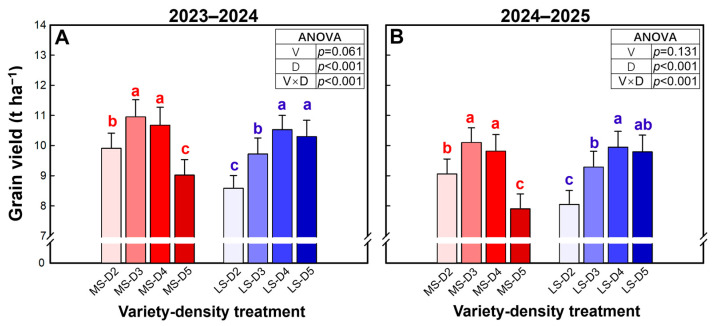
Grain yield of winter wheat in the 2023–2024 (**A**) and 2024–2025 (**B**) growing seasons. Data are means, and error bars are SD (n = 4). Different lowercase letters indicate that there are significant differences among the densities for the specific variety according to the least significant difference test (*α* = 0.05). MS, mitule-spike type; LS, large-spike type; D2, D3, D4, and D5 indicate 200, 300, 400, and 500 plants m^−2^, respectively.

Grain yield was significantly affected by density and by the variety × density interaction in both seasons, whereas the main effect of variety was not significant (Figure 1). Averaged across densities, MS yielded 10.16 and 9.25 t ha^−1^ in 2023–2024 and 2024–2025, respectively, while LS yielded 9.81 and 9.03 t ha^−1^. Across the two growing seasons, MS showed a pattern in which grain yield increased significantly with increasing plant density, then remained relatively stable, and finally declined significantly at the highest density, with the highest yield of 10.13–10.97 t ha^−1^ occurring at D3; whereas LS exhibited a continuous increase in yield followed by a slight but non-significant decrease when density was increased to D4, with the maximum yield of 9.97–10.55 t ha^−1^ occurring at D4.

Averaged across plant densities, MS exhibited 26.4–27.0% higher maximum stem number and 25.7–26.6% higher productive stem percentage than LS (Table 1). In both cultivars, the maximum stem number increased significantly with increasing density, whereas the productive stem percentage showed a decreasing trend. Spike number was 60.2–60.9% higher in MS than in LS, while grains per spike and grain weight were 28.4–29.0% and 9.6–12.3% lower in MS, respectively. Consistent with these patterns, spike number increased significantly with increasing density in both cultivars, whereas grains per spike and grain weight declined. Because yield per spike is the product of grain per spike and grain weight, it also decreased markedly as density increased. LS produced 53.1–59.1% greater spike yield than MS on average. By contrast, grain number displayed a distinct density response between cultivars: under MS, it increased significantly at lower densities and then decreased significantly at higher densities, peaking with 24.0–24.2 × 10^3^ m^–2^ at D4; whereas under LS, it increased continuously with increasing density, with the greatest value of 21.7–21.9 × 10^3^ m^–2^ at D5. Regarding canopy traits, MS had a 4.5–5.6% higher LAI than LS on average (*p* < 0.05), but the ratio of leaf area to grain number (LA/G) was 7.2–7.3% lower. In both cultivars, LAI increased consistently with increasing density; however, the density response of LA/G differed between cultivars, showing a clear increasing trend under MS but no significant trend under LS.

### 2.2. Dry Matter Production and Harvest Index

**Figure 2:**
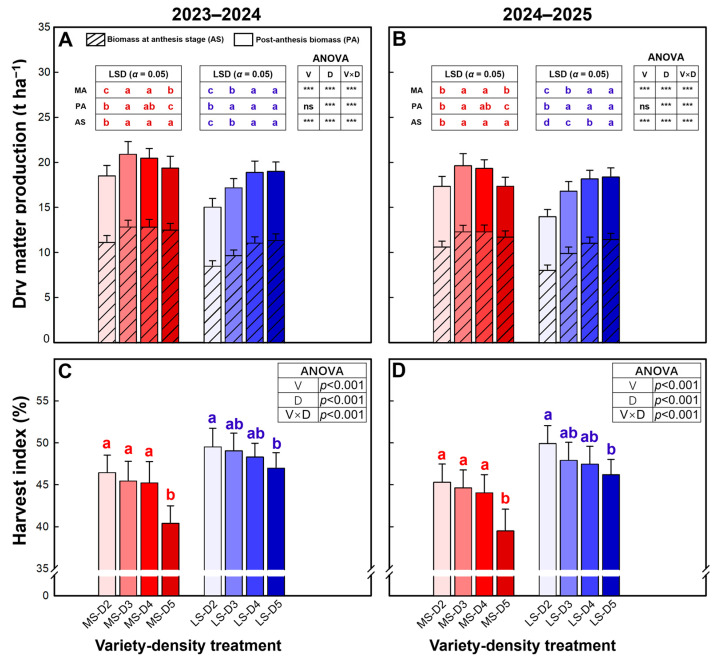
Dry matter production (**A**,**B**) and harvest index (**C**,**D**) of winter wheat in the 2023–2024 and 2024–2025 growing seasons. Data are means, and error bars are SD (n = 4). Different lowercase letters indicate that there are significant differences among the densities for the specific variety according to the least significant difference test (*α* = 0.05). AS, dry matter at anthesis stage; PA, post-anthesis dry matter; MA, dry matter at maturity; MS, mitule-spike type; LS, large-spike type; D2, D3, D4, and D5, 200, 300, 400, and 500 plants m^−2^, respectively; *** indicates significance at *p* = 0.001, and ns indicates non-significance at *p* = 0.05.

Dry matter production was significantly affected by variety, plant density, and their interaction, except that post-anthesis dry matter was not significantly influenced by variety (Figure 2A,B). Overall, MS accumulated more biomass than LS: at anthesis, MS produced 15.9–21.2% more dry matter on average, which translated into a 9.3–12.9% higher total aboveground dry matter at maturity. The density response of anthesis dry matter also differed markedly between varieties. In MS, anthesis dry matter increased initially with increasing density and then remained stable, peaking at D3 or D4, with a maximum increase of 1683–1708 kg ha^−1^. By contrast, LS showed a continuous increase in anthesis dry matter with increasing density, reaching the maximum at D5 and exhibiting a much larger maximum increase of 2.86–3.42 t ha^−1^. Post-anthesis dry matter accumulation in MS increased significantly with density and remained relatively high at D3 and D4 but declined significantly at D5, being 14.7–23.3% lower than the maximum observed at D3. In LS, post-anthesis dry matter increased significantly and then plateaued at a relatively high level; the maximum occurred at D4, and the slight decrease at D5 relative to D4 was not significant (2.4–3.0%). Consistently, total aboveground dry matter at maturity peaked at D3 in MS, with D5 being 7.3–11.5% lower than D3, whereas LS reached the maximum at D5, which was 9.3–10.5% higher than D2.

The harvest index was significantly affected by variety, plant density, and their interaction (Figure 2C,D). On average, the harvest index was 9.1–10.3% higher in LS than in MS. In MS, the harvest index did not differ significantly among D2–D4 but was significantly higher than that at D5. In LS, the harvest index showed a slight decreasing trend with increasing density, and a significant reduction occurred only between D2 and D5 (5.1–7.4%).

### 2.3. Relationship Between Yield and Sink Size and Source Capacity

**Figure 3:**
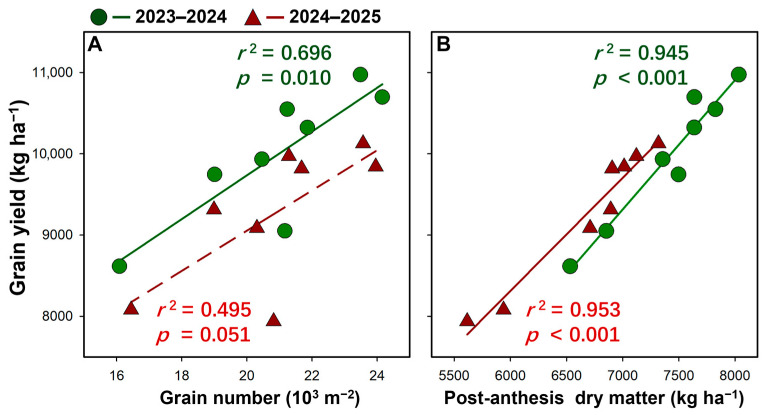
Relationship between yield and grain number (**A**) and post-anthesis dry matter production (**B**) in the 2023–2024 and 2024–2025 growing seasons.

Grain yield was positively related to sink size (grain number) in 2023–2024, with a significant linear correlation (*r*^2^ = 0.696, *p* = 0.010; Figure 3A). In 2024–2025, the yield–grain number relationship was weaker and insignificant (*r*^2^ = 0.495, *p* = 0.051). By contrast, grain yield showed a strong positive linear relationship with source capacity, represented by post-anthesis dry matter production, in both seasons (*r*^2^ = 0.945–0.953, *p* < 0.001; Figure 3B). Overall, these results suggest that yield variation was more tightly associated with post-anthesis dry matter production.

### 2.4. Radiation Interception and Utilization

Intercepted solar radiation (ISR) during the pre-anthesis phase and during the whole season was significantly affected by variety, and ISR was significantly impacted by density in both seasons (Table 2). Averaged across densities, MS intercepted 17.2–21.7% higher pre-anthesis and 10.0–12.2% total ISR than LS. Increasing density significantly increased intercepted radiation in both varieties; the highest total ISR occurred at the highest density (D5), reaching 2069.3 and 2104.6 MJ m^−2^ in MS and 1878.1 and 1955.5 MJ m^−2^ in LS in 2023–2024 and 2024–2025, respectively.

RUE was strongly affected by density and the variety × density interaction for pre-anthesis, post-anthesis, and seasonal values, while the main effect of variety on RUE was not significant (Table 2). For MS, RUE declined markedly with increasing density, with pre-, post-anthesis, and seasonal RUE decreasing by 14.5–18.1%, 16.5–23.7%, and 14.8–19.4%, respectively, from D2 to D5. In contrast, LS maintained relatively stable RUE across densities, except for only a 3.7–4.7% (*p* < 0.05) reduction in post-anthesis RUE from D2 to D5.

### 2.5. N Uptake and Efficiencies of N Use

**Figure 4:**
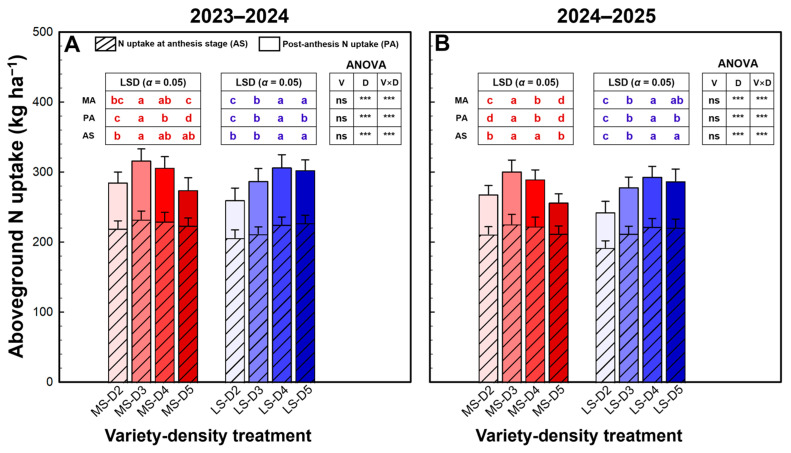
Aboveground N uptake of winter wheat in the 2023–2024 (**A**) and 2024–2025 (**B**) growing seasons. Data are means, and error bars are SD (n = 4). Different lowercase letters indicate that there are significant differences among the densities for the specific variety according to the least significant difference test (*α* = 0.05). AS, N uptake at anthesis stage; PA, post-anthesis N uptake; MA, N uptake at maturity; MS, mitule-spike type; LS, large-spike type; D2, D3, D4, and D5, 200, 300, 400, and 500 plants m^−2^, respectively; *** indicates significance at *p* = 0.001, and ns indicates non-significance at *p* = 0.05.

Aboveground N uptake at maturity and its components—N uptake at anthesis and post-anthesis N uptake—were significantly affected by density and the variety × density interaction in both seasons, whereas the main effect of variety was not significant (Figure 4). In MS, total N uptake increased from D2 to D3 and then declined, declining at D5 to a level similar to D2. This pattern was mainly driven by post-anthesis N uptake, which peaked at D3 and decreased markedly at D5, while N uptake at anthesis increased to D3 (and remained comparable at D4) but did not continue to rise at the highest density. In contrast, LS exhibited a more positive response to increasing density: pre-anthesis N uptake increased from D2 to D4, and D4 was statistically similar to D5 in both seasons, accompanied by consistently higher post-anthesis N uptake at the higher densities, resulting in the highest level of total N uptake at D4.

**Figure 5:**
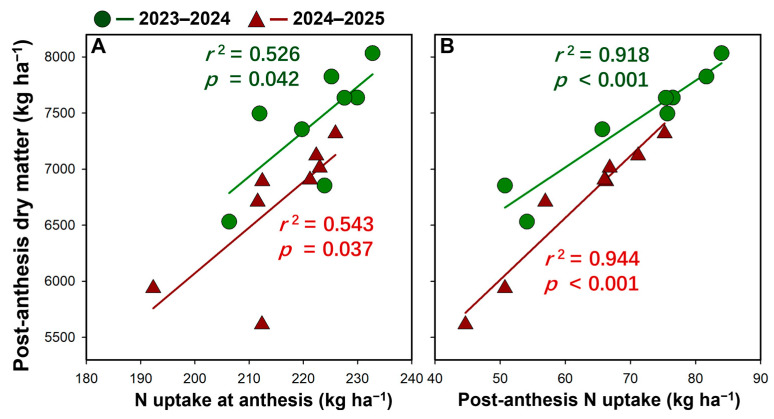
Relationship between post-anthesis dry matter and N uptake at anthesis (**A**) and after anthesis (**B**) in the 2023–2024 and 2024–2025 growing seasons.

Post-anthesis dry matter accumulation was positively correlated with N uptake (Figure 5). Specifically, post-anthesis dry matter increased significantly with increasing N uptake at anthesis in both seasons (0.543 ≥ *r*^2^ ≥ 0.526, 0.037 ≤ *p* ≤ 0.042). An even stronger relationship was observed between post-anthesis dry matter and post-anthesis N uptake, with highly significant linear correlations in both seasons (0944 ≥ *r*^2^ ≥ 0.918, *p* < 0.001).

**Figure 6:**
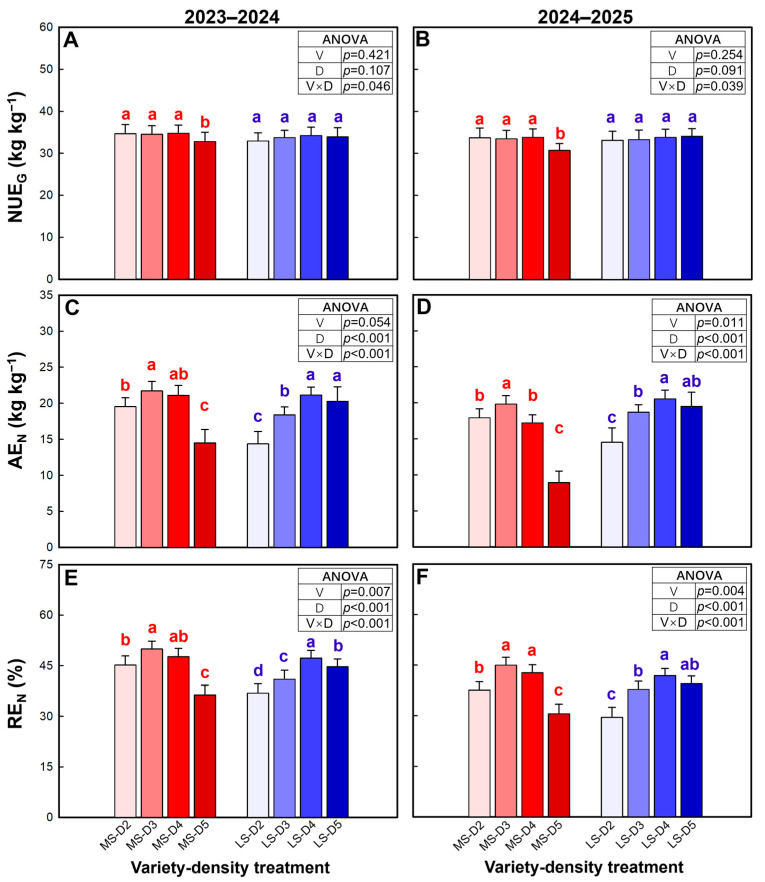
Nitrogen use efficiency for grain production (NUE_G_, (**A**,**B**)), agronomic efficiency of N fertilizer (AE_N_, (**C**,**D**)), and recovery efficiency of N fertilizer (RE_N_, (**E**,**F**)) in the 2023–2024 and 2024–2025 growing seasons. Data are means, and error bars are SD (n = 4). Different lowercase letters indicate that there are significant differences among the densities for the specific variety according to the least significant difference test (*α* = 0.05). MS, mitule-spike type; LS, large-spike type; D2, D3, D4, and D5, 200, 300, 400, and 500 plants m^−2^, respectively.

NUE_G_ (N use efficiency for grain production) was not significantly affected by variety or density in either season (Figure 6A,B). However, the variety × density interaction was significant in both seasons, largely because MS exhibited a significant reduction in NUE_G_ at the highest density (D5) relative to D2–D4, while LS maintained relatively stable NUE_G_ across all density treatments.

AE_N_ (agronomic use efficiency of fertilizer N) was strongly influenced by density and the variety × density interaction in both seasons, while the main effect of variety was marginal in 2023–2024 and significant in 2024–2025 (Figure 6C,D). In MS, AE_N_ increased to a maximum at D3 and then declined, with D5 being 32.9–54.4% lower than D2. In contrast, LS showed a generally increasing response to density, peaking at D4 (and remaining statistically comparable at D5), indicating a medium-to-high density plateau.

RE_N_ (recovery efficiency of fertilizer N) was significantly affected by variety, density, and their interaction in both seasons (Figure 6E,F). In MS, RE_N_ increased at intermediate densities, peaking at D3 and remaining stable at D4, but dropped sharply at D5 (by 26.0–31.7% relative to D3). In LS, RE_N_ increased markedly with density and reached its maximum at D4, with only a slight reduction (5.4–5.7%) at D5, although this decrease was significant in 2023–2024.

### 2.6. N Nutrition Index

**Figure 7:**
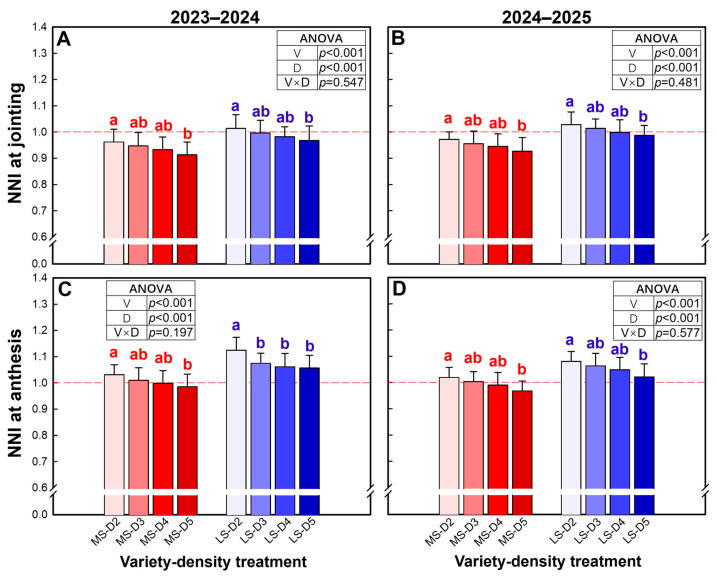
Nitrogen nutrition index (NNI) at joining stage (**A**,**B**) and anthesis stage (**C**,**D**) in the 2023–2024 and 2024–2025 growing seasons. Data are means, and error bars are SD (n = 4). Different lowercase letters indicate that there are significant differences among the densities for the specific variety according to the least significant difference test (*α* = 0.05). MS, mitule-spike type; LS, large-spike type; D2, D3, D4, and D5, 200, 300, 400, and 500 plants m^−2^, respectively.

The N nutrition index (NNI) at both jointing and anthesis was significantly affected by variety and plant density (Figure 7). At the jointing stage, LS exhibited a 5.4–6.0% higher mean NNI than MS (Figure 7A,B). In both varieties, NNI generally declined with increasing density, with the highest values at D2, the lowest at D5, and intermediate values at D3–D4. Notably, in LS, NNI at D2 and D3 at jointing remained consistently around or slightly above 1.0 in both seasons, whereas all other treatments were clearly below 1.0. At anthesis, a similar density-dependent decrease was observed (Figure 7C,D), and LS maintained a 5.8–7.2% higher mean NNI than MS. Specifically, NNI at anthesis fell clearly below 1.0 only in MS at D5, while it was around or above 1.0 in all other treatments.

**Figure 8:**
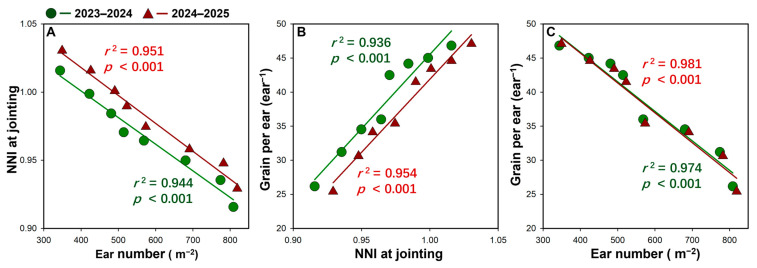
Relationships among NNI at jointing, spike number, and grain per spike across varieties and densities in the 2023–2024 and 2024–2025 growing seasons.

NNI at jointing was closely associated with variation in yield components. Spike number showed a strong negative linear relationship with NNI at jointing in both seasons (*r*^2^ ≥ 0.944, *p* < 0.001, Figure 8A), indicating that more spikes were accompanied by a lower N nutritional status. In contrast, grains per spike were positively correlated with NNI at jointing (*r*^2^ ≥ 0.936, *p* < 0.001, Figure 8B). Consistently, grain per spike declined markedly as spike number increased, showing a strong negative relationship in both seasons (*r*^2^ ≥ 0.974, *p* < 0.001, Figure 8C). Together, these results suggest that increased spike number tended to reduce NNI at jointing, which in turn was associated with fewer grains per spike.

### 2.7. Soil Water Consumption and Water Use Efficiency

**Figure 9:**
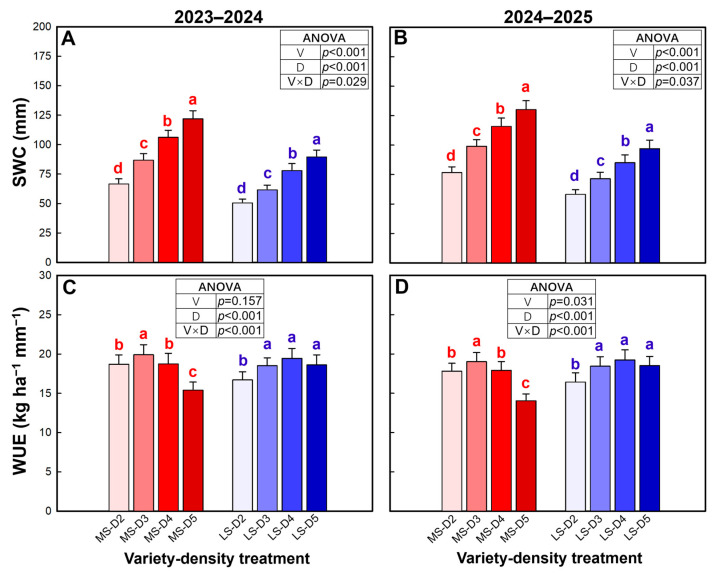
Soil water consumption (SWC, (**A**,**B**)) and water use efficiency (**C**,**D**) in the 2023–2024 and 2024–2025 growing seasons. Data are means, and error bars are SD (n = 4). Different lowercase letters indicate that there are significant differences among the densities for the specific variety according to the least significant difference test (*α* = 0.05). MS, mitule-spike type; LS, large-spike type; D2, D3, D4, and D5, 200, 300, 400, and 500 plants m^−2^, respectively.

Soil water consumption (SWC) was significantly affected by variety, density, and their interaction in both seasons (Figure 9A,B). MS increased the average SWC by 34.8–36.0% over LS. Across densities, MS consistently showed greater SWC than LS, and the magnitude of the density-driven increase was larger in MS, resulting in a significant variety × density interaction. SWC increased progressively with increasing density in both varieties, reaching the highest at D5.

Water use efficiency (WUE) was strongly influenced by density and the variety × density interaction in both seasons, while the main effect of variety was not significant in 2023–2024 (*p* = 0.157, Figure 9C) but became significant in 2024–2025 (*p* = 0.031, Figure 9D). In MS, WUE increased from D2 to a maximum at D3 and then declined with further increases in density, with a pronounced reduction (of 22.5–25.9%) at D5 relative to D2. In contrast, LS showed an increasing trend in WUE from D2 to D4 and then maintained a relatively high plateau at D4–D5, with only a slight and insignificant decrease (3.7–4.2%) at the highest density.

## 3. Discussion

### 3.1. Effects of Variety and Density on Yield and Its Components

The present study demonstrates that, even under drip fertigation with relatively enhanced water–N supply, optimal plant density remains strongly variety-dependent when varieties differ in individual spike productivity. The most important finding is the consistently significant variety × density interaction across grain yield and multiple physiological traits, indicating that density optimization cannot be generalized across varieties—a conclusion that agrees with evidence that agronomic optimum density varies with genetic background and yield environment [2,3,4]. Our previous research showed that, across a wide range of planting densities, wheat grain yield exhibits a strong quadratic response to density [8]. Consistently, the present study also found that the yield–density relationships of both varieties generally followed a quadratic function; however, the optimal density differed markedly between the two varieties. The MS variety exhibited a clear “increase–plateau–decline” yield pattern as density increased, with yield penalties emerging at the highest density, whereas the LS variety showed a more persistent yield increase and only a slight (often non-significant) reduction at the highest density. These contrasting responses support a trait-based strategy in drip-fertigated wheat: varieties that rely more on stand establishment and tillering compensation require a different density target than varieties that depend more on individual spike productivity and benefit from higher baseline spike number. The contrasting yield responses of MS and LS to planting density may be largely attributed to their differences in tillering capacity and spike development. MS relied more heavily on spike number formation and therefore responded more sensitively to density-induced changes in tillering, whereas LS showed greater compensatory capacity through spike development, which contributed to its relatively stable yield across densities.

Many studies have shown that excessively high plant density usually leads to a substantial increase in tiller number and spike number, and the intensified inter-plant competition can impair grain filling, thereby reducing grain weight and individual plant (or spike) productivity [46,47,48,49,50]. However, other studies have indicated that varieties differing in individual spike productivity may exhibit markedly different magnitudes and underlying mechanisms in their yield responses to agronomic management practices [20,26,27,51,52]. The divergent yield responses were primarily explained by differences in yield-component coordination and by how density reshaped the balance between spike number and individual spike performance. Across both varieties, increasing density increased spike number but reduced grains per spike and grain weight; consequently, yield per spike declined as density increased. However, LS maintained a stronger advantage in spike yield, and its grain number continued to rise with density, whereas MS showed a unimodal grain-number response, increasing at lower densities but decreasing at higher densities. This indicates that MS gained yield mainly through increasing sink size up to an intermediate density, but its sink formation became constrained at higher densities. In contrast, LS continued to strengthen sink size through spike number formation at higher density, allowing it to sustain grain number and yield. The canopy trait LA/G further clarifies this difference. Although LS had a higher LAI, its lower LA/G suggests a more efficient leaf area allocation per grain, whereas MS showed a pronounced increase in LA/G with density, implying that the additional leaf area generated at higher density was not matched by proportional gains in grain number. Such a mismatch is consistent with intensified intra-canopy competition and diminished efficiency of converting canopy development into effective sink formation under MS at high density.

### 3.2. Effects of Variety and Density on Dry Matter Production and Partitioning

The final crop yield is jointly determined by dry matter accumulation and the efficiency of its partitioning to the grains (i.e., harvest index) [17,18,19]. Particularly, the post-anthesis dry matter plays a central role in determining wheat yield differences among treatments [20,21,53]. In the present study, dry matter production provided stronger mechanistic support for a source–sink framework. MS produced substantially more dry matter at anthesis and maintained higher total aboveground dry matter at maturity than LS, yet its harvest index was lower, indicating that biomass advantages did not fully translate into grain yield under all densities. Especially at high density, MS showed a marked decline in harvest index (Figure 2C,D), indicating that, for MS, excessive density not only restricted dry matter accumulation but also reduced the efficiency of dry matter partitioning to the grain. Moreover, we observed in the field that the highest density (D5) caused varying degrees of localized lodging in some plots, posing a major threat to yield stability. Density responses of dry matter further revealed that MS peaked earlier (low-to-intermediate density) and then declined at higher density, whereas LS increased more consistently and reached its maximum total dry matter at higher density. Importantly, grain yield was more tightly associated with post-anthesis dry matter production than with grain number (Figure 3), especially in 2024–2025, highlighting that yield formation under drip fertigation was strongly governed by the capacity to maintain carbon assimilation and biomass accumulation during grain filling. Similar results were also reported by Lu et al. using a large spike variety in Shandong province, China [20]. The density-induced decline in MS post-anthesis dry matter at high density therefore provides a direct explanation for its yield reduction and for the concurrent declines in grain weight and harvest index.

Dry matter production results from canopy interception of solar radiation and its conversion into chemical energy stored in biomass [22,23,24,26]. Therefore, it is essential to analyze the effects of variety and plant density, as well as their interaction, on these two key processes (radiation interception and ISR and radiation use efficiency, RUE). The classic crop physiological framework established that biomass accumulation can be interpreted as the product of intercepted radiation and RUE, which provides a useful basis for dissecting density responses. Numerous studies have shown that increasing plant density generally enhances canopy ISR by increasing canopy cover and leaf area development, but excessive density may also intensify canopy shading and worsen within-canopy light distribution, thereby causing varying degrees of decline in RUE (trade-off between ISR and RUE) [5,6,25,54]. In the present study, the contrasting density responses of MS and LS can be interpreted as a trade-off between radiation capture and radiation conversion efficiency. Increasing density from D2 to D5 consistently increased canopy radiation interception in both varieties, and the highest total ISR occurred at D5 in both seasons. Moreover, averaged across densities, MS intercepted 17.2–21.7% more pre-anthesis ISR and 10.0–12.2% more total ISR than LS, indicating a stronger canopy radiation-capture capacity. However, this ISR advantage did not translate into a yield advantage at high density because RUE in MS declined markedly with increasing density: from D2 to D5, pre-anthesis, post-anthesis, and seasonal RUE decreased by 14.5–18.1%, 16.5–23.7%, and 14.8–19.4%, respectively. We suggest that under high planting density, the denser canopy structure of MS likely intensified intra-canopy shading, reduced light penetration into the middle and lower canopy, and accelerated leaf senescence, especially in shaded leaves. As a result, although radiation interception may not have decreased substantially, the efficiency of converting intercepted radiation into biomass was likely reduced, leading to the observed decline in RUE. In contrast, LS maintained relatively stable RUE across densities, with only a small but significant decline in post-anthesis RUE (3.7–4.7%) from D2 to D5. These results indicate that, in MS, higher density increased radiation interception but simultaneously weakened the efficiency of converting intercepted radiation into biomass (especially after anthesis), thereby contributing to reduced post-anthesis dry matter production, lower harvest index, and yield penalties at the highest density. By contrast, LS was able to combine increased ISR with a much smaller RUE penalty, which helps explain why its yield remained high and relatively stable at medium-to-high densities.

### 3.3. Effects of Variety and Density on N Uptake, Nutrition Index, and Use Efficiencies

The nitrogen nutrition index (NNI) provides an important physiological bridge linking density regulation to yield formation and resource-use efficiency in the present study. NNI is widely recognized as a robust in-season indicator of crop N nutritional status because it evaluates plant N status relative to the critical N concentration required to sustain maximum biomass growth at a given biomass level, thereby accounting for the ontogenetic decline (dilution) in plant N concentration as canopy biomass increases [32,33,55]. This makes NNI particularly suitable for comparing N status across varieties and plant densities, where biomass accumulation and N demand differ markedly. In our study, NNI declined with increasing density at both jointing and anthesis in both varieties, indicating progressively stronger N competition as population size increased; however, LS maintained a consistently higher NNI than MS, and NNI remained around or slightly above 1.0 at jointing under D2–D3 in LS, whereas most other treatments were below 1.0. At anthesis, NNI fell clearly below 1.0 only in MS at D5, suggesting that excessive density imposed a stronger N limitation on MS during a critical period for sink establishment and grain filling. This interpretation is further supported by the strong relationships among NNI and yield components: spike number was negatively associated with NNI at jointing, whereas grains per spike were positively associated with NNI, and grains per spike declined sharply as spike number increased (Figure 8). Together, these results indicate that density-driven increases in spike number were achieved at the expense of crop N nutritional status, which in turn constrained per-spike sink size. Importantly, this N status effect was closely coupled with post-anthesis source activity and overall resource-use performance. In MS, the decline in NNI at high density coincided with reduced post-anthesis N uptake, lower post-anthesis dry matter accumulation, and marked reductions in RUE, AE_N_, and RE_N_, indicating a coordinated collapse in resource capture and conversion under excessive density. By contrast, LS maintained higher NNI, stronger N uptake (especially at anthesis and during grain filling), and relatively stable RUE at medium-to-high densities, which likely helped sustain post-anthesis dry matter production and yield stability. During grain filling, rapid carbohydrate deposition proceeds concurrently with grain protein synthesis [56], and grain N is supplied by both post-anthesis soil N uptake and remobilization of stored N, including photosynthetic N in leaves [57]. Therefore, the greater post-anthesis N uptake maintained by LS may have reduced the need for remobilization of leaf photosynthetic N to support grain protein formation, which might partly explain its relatively greener canopy and higher photosynthetic capacity. By contrast, the decline in post-anthesis N uptake in MS at high density may have increased the requirement for leaf N remobilization, potentially contributing to reduced source activity.

### 3.4. Effects of Variety and Density on Water Consumption and Use Efficiency

Although increasing plant density can enhance crop N uptake, canopy radiation interception, and dry matter accumulation by strengthening population size and canopy development, it also increases crop water consumption, and excessive density may ultimately reduce WUE when the gain in grain yield fails to compensate for the increase in evapotranspiration. Planting density may affect WUE not only through changes in total water consumption but also through its influence on the partitioning of water use between transpiration and soil evaporation. Increasing density generally enhances canopy coverage and reduces soil evaporation, which may improve WUE when a greater proportion of water is used for transpiration that directly supports biomass accumulation. However, excessively high density may also increase canopy transpiration demand and intensify intra-canopy shading and competition, thereby reducing the efficiency with which water is converted into biomass and grain yield. In the present study, this mechanism may partly account for the relatively low WUE observed at D5 in MS. This trade-off is also well supported by previous studies in wheat. For example, Ma et al. reported that, under the same sowing-time condition, soil water consumption increased significantly with density and WUE decreased significantly with increasing density in some sowing-time treatments, indicating that excessive stand density can aggravate “growth redundancy” and reduce water productivity [37]. Similarly, Hu et al. showed that wheat field evapotranspiration was higher under medium and high densities than under low rates and that low density reduced ET and tended to increase WUE, particularly when high density accelerated water depletion during vegetative growth and reduced harvest index [58]. However, the effect of density on water productivity is not universally negative: under limited irrigation, increasing density can improve root length density in deeper soil layers, enhance deep soil moisture uptake, and improve water productivity when population architecture remains favorable. Therefore, the water-use consequence of density regulation should be interpreted as a variety- and environment-dependent balance between increased water consumption and the efficiency of converting that water into grain, rather than a simple monotonic effect. This interpretation is consistent with broader evidence that wheat responses to plant density depend on yield environment and tillering potential and with crop physiological analyses showing that WUE is tightly coupled with canopy radiation-use processes (RUE, ISR, and canopy structure).

This study demonstrates that, under drip fertigation, even with a more stable water–N supply, the optimal plant density of wheat remains strongly dependent on variety type (particularly individual spike productivity), rather than being amenable to a single uniform density recommendation. The contrasting density responses of the two varieties were essentially driven by differences in the integrated mechanism of spike number formation, individual spike productivity, source–sink coordination, and coordinated resource-use efficiency. In MS, higher density increased population size and radiation interception, but it also led to declines in RUE, post-anthesis N uptake, NNI, and post-anthesis dry matter accumulation, accompanied by reductions in HI, AE_N_, RE_N_, and WUE, ultimately causing yield loss at excessive density. By contrast, LS was better able to secure spike number at medium-to-high densities while maintaining relatively stable RUE, N nutritional status, and water-use efficiency, thereby sustaining high and stable yield. These findings suggest that density optimization for drip-fertigated wheat should be based on individual spike productivity traits and their associated nitrogen–radiation–water coordinated efficiency and implemented through a variety-specific precision management strategy.

## 4. Materials and Methods

### 4.1. Site Description

**Figure 10:**
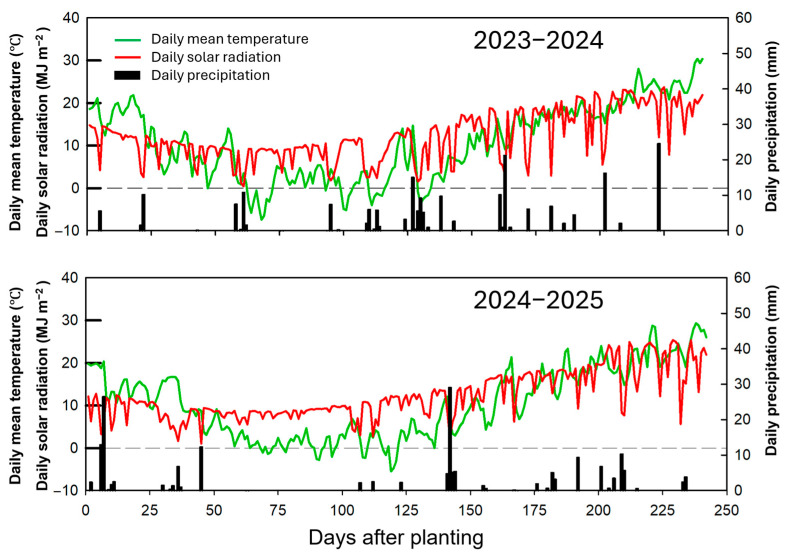
Daily climatic conditions in the 2023–2024 and 2024–2025 growing seasons.

Two consecutive field experiments were conducted during the 2023–2024 and 2024–2025 winter wheat growing seasons in Quanyuan Town, Tancheng County, Shandong Province, China (34°42′ N, 118°25′ E). The region is characterized by a winter wheat–summer maize rotation as the dominant cropping system. Seasonal precipitation was 221.9 mm in 2023–2024 and 190.8 mm in 2024–2025 (Figure 10). The experimental soil is locally classified as a fluvo-aquic soil with a clay loam texture, which is widely distributed in the winter wheat–summer maize production region of northern China. Before sowing each season, soil samples from the 0–20 cm layer were collected to determine initial fertility status. In 2023 and 2024, respectively, soil organic matter was 14.6 and 15.1 g kg^−1^, total N was 0.88 and 0.85 g kg^−1^, Olsen-P was 17.56 and 17.94 mg kg^−1^, available K was 125.6 and 129.2 mg kg^−1^, and pH was 8.14 and 8.12. Soil pH was determined in H_2_O using a soil-to-solution ratio of 1:2.5.

### 4.2. Experimental Design and Crop Management

The experiment was conducted in a split-plot design with four replications, with variety (V) assigned to the main plots and plant density (D) randomized within each main plot. Each subplot measured 7.5 m × 1.6 m and comprised eight rows with a 0.20 m row spacing. Two high-yielding winter wheat varieties contrasting in spike type were used: Jimai23 (multi-spike type; MS) and Jimai24 (large-spike type; LS). Four target plant densities were established: 200, 300, 400, and 500 plants m^−2^ (D2, D3, D4, and D5, respectively).

Plots were established by manual sowing at an approximate depth of 3 cm on 14 October 2023 and 12 October 2024. To ensure accurate achievement of target densities, seeds were sown at 1.25 times the target density, and seedlings were then hand-thinned at the stage when the second leaf was fully expanded, resulting in the intended density and a uniform spatial distribution.

Phosphorus and potassium were uniformly applied to all plots before sowing at rates of 120 kg P_2_O_5_ ha^−1^ (as calcium superphosphate, 16% P_2_O_5_) and 90 kg K_2_O ha^−1^ (as potassium chloride, 52% K_2_O), respectively. Nitrogen was applied at a total rate of 250 kg N ha^−1^ (as urea, 46% N), including 100 kg N ha^−1^ as a basal application and the remaining 150 kg N ha^−1^ supplied through fertigation in four splits: 25, 75, 25, and 25 kg N ha^−1^ at green-up, jointing, booting, and anthesis, respectively. The N fertilizer rate applied in this study was determined based on previous studies conducted under similar climatic conditions or at the same experimental site [41,42,43,50] and was chosen to reflect the N application levels commonly used in local winter wheat production.

Irrigation was applied using a drip system six times each season: before winter dormancy and at green-up, jointing, booting, anthesis, and early milk stage, with 40 mm applied at each event. Drip tapes (16 mm diameter) were installed at 0.40 m spacing, with one tape serving two wheat rows. Emitters were spaced 0.30 m apart with a nominal discharge rate of 1 L h^−1^. During fertigation, urea was dissolved and injected into the irrigation line using a Venturi injector, then mixed before delivery to each plot. Each fertigation event lasted approximately 6 h: fertilizer injection began in the second hour and continued for about 3 h, followed by flushing with clean water to clear the lines.

To calculate fertilizer-N recovery efficiency (RE_N_) and agronomic efficiency (AE_N_), additional no-N control plots for all four densities under both varieties were established in a nearby field, which had long been managed under the same cropping system and agronomic practices as the main experimental site, and were maintained under the same management conditions as the experimental plots except for N application.

All plots were managed to minimize confounding stress effects. Diseases and insect pests were controlled as required, and weeds were suppressed by herbicide application three times per growing season.

### 4.3. Sampling and Measurements

The maximum stem number (main stems + tillers, MSN) was recorded at the jointing stage. Stem counts were made in four representative central rows over a length of 1.25 m, corresponding to 1.0 m^2^. At maturity, the productive spike number per unit spike (EN) was determined, and the productive stem percentage (PSP) was calculated as the ratio of spike number at maturity to the maximum stem number at jointing. The productive spike produced no less than five grains.

Canopy development was evaluated at anthesis. Plants were sampled from a representative central row over 0.50 m (equivalent to 0.100 m^2^). Green leaf blades were separated, and leaf area was measured using a leaf area meter (LI-3100C, LI-COR, Lincoln, NE, USA). Leaf area index (LAI) was calculated as leaf area divided by sampled ground area.

For dry matter and plant N analyses, all aboveground plants were collected at anthesis and physiological maturity (Zadoks 89) from a 0.50 m segment of a central row (0.100 m^2^) in each plot. At anthesis, samples were separated into stems (including sheaths), leaf blades, and spikes; at maturity, samples were divided into straw and grain. Plant materials were first heated at 105 °C for 30 min and then oven-dried at 80 °C to constant weight for dry matter determination. Tissue N concentration (mg g^−1^) was determined using an automated Kjeldahl analyzer (Kjeltec 8400, FOSS, Hillerød, Denmark). N uptake was calculated by multiplying aboveground dry matter by plant N concentration.

Based on yield and N uptake data, N use efficiency for grain production (NUE_G_), agronomic efficiency of fertilizer N (AE_N_), and fertilizer N recovery efficiency (RE_N_) were calculated as follows:

(1) $${\mathrm{NUE}}_{G}\;\left({\mathrm{kg}\;\mathrm{kg}}^{-1}\right)=\mathrm{Yield}\;/\;N\;\mathrm{uptake}$$

(2) $${\mathrm{AE}}_{N}\;\left({\mathrm{kg}\;\mathrm{kg}}^{-1}\right)=\left(\mathrm{Yield}-Y_{0}\right)\;/\;N\;\mathrm{rate}$$

(3) $${\mathrm{RE}}_{N}\;\left(\%\right)=\left(N\;\mathrm{uptake}-N_{0}\right)\;/\;N\;\mathrm{rate}\;\times 100$$

where N uptake is total aboveground N uptake at maturity; Y_0_ and N_0_ are grain yield and N uptake in the zero-N control plots, respectively; and N rate is the applied N rate.

Crop N nutritional status at anthesis was assessed using the nitrogen nutrition index (NNI), defined as the ratio of actual aboveground N concentration (Nt) to the critical N concentration (Nct):

(4) $$\mathrm{NNI}=N_{t}\;/\;N_{\mathrm{ct}}$$

The critical N concentration (N_ct_) was estimated using the wheat critical N dilution equation proposed by Justes et al. [33] as a function of aboveground dry matter at anthesis (DM):

(5) $$N_{\mathrm{ct}}=5.35\times {\mathrm{DM}}^{-0.442}$$

The actual N concentration at anthesis (N_t_) was calculated as N uptake divided by aboveground dry matter at anthesis.

At maturity, grain yield was measured by harvesting spikes from the central area of each plot over 2.0 m^2^ (2.0 m in length across five representative rows). Yield was adjusted to a standard grain moisture content of 13.0%, which was measured using a digital moisture tester (PM8188A, Kett Electric Laboratory, Tokyo, Japan). Single-grain weight (GW) was determined from three randomly selected 50.00 g grain subsamples and was also corrected to 13.0% moisture. Grains per spike (GPS) were calculated as follows:

(6) $$\mathrm{GPS}=\mathrm{Yield}\;/\;\left(\mathrm{GW}\times \mathrm{EN}\right)/1000$$

where Yield is grain yield per unit area (g m^−2^), GW is single-grain weight (mg), and EN is spike number per m^2^.

Canopy photosynthetically active radiation (PAR) interception was monitored throughout the growing seasons. Measurements were taken every 7–15 days between 1100 and 1300 h using a linear PAR ceptometer (AccuPAR LP-80, Decagon Devices Inc., Pullman, WA, USA). In each plot, transmitted PAR (PAR_t_) was measured by placing the light bar perpendicular to the crop rows and just above the soil surface, and PAR above the canopy (PAR_a_) was recorded immediately afterward. Six paired measurements (below and above the canopy) were taken in each plot. The canopy PAR interception ratio (PARI) was calculated as follows:

(7) $$\mathrm{PARI}\;\left(\%\right)=\left({\mathrm{PAR}}_{a}-{\mathrm{PAR}}_{t}\right)/{\;\mathrm{PAR}}_{a}\;\times 100$$

Intercepted solar radiation (ISR) during each growth period was estimated using the average canopy PAR interception ratio (PARI) over that period, together with the accumulated incident solar radiation for the same interval. Seasonal ISR was then obtained by summing the ISR values across all growth periods. Radiation use efficiency (RUE) for a given period was determined as the amount of biomass produced per unit of intercepted solar radiation during that period. Seasonal ISR was obtained by summing the intercepted solar radiation values across all growth periods. RUE for each period was expressed as dry matter production per unit of ISR during that period.

Soil water was measured before sowing and after maturity using a 5 cm-diameter auger. Samples were taken from the 0–200 cm profile at 20 cm intervals (10 layers in total). Fresh soil samples were immediately sealed in aluminum boxes and transferred to the laboratory for determination of gravimetric water content by the oven-drying method. These measurements were used to estimate soil water consumption (SWC) in the 0–200 cm layer during the growing season and to calculate crop evapotranspiration (ET).

Crop evapotranspiration (ET, mm) was calculated as follows:

(8) ET=I + P− R− D + SWC

where I is irrigation (mm), P is precipitation (mm), R is surface runoff (mm), D is deep drainage (mm), and SWC is the consumption of soil water storage (mm) in the 0–200 cm profile between pre-sowing and post-harvest sampling. In this study, R and D were considered negligible because the field surface was flat and infiltration did not exceed the monitored 0–200 cm soil depth.

The calculation methods used in Equations (1)–(8) were adopted from previous studies [37,42,45].

Water use efficiency (WUE, kg ha^−1^ mm^−1^) was expressed as grain yield divided by ET.

### 4.4. Data Analysis

Statistical analyses were performed with Statistix 9.0 (Analytical Software, Tallahassee, FL, USA). Data were analyzed separately for each season because the two growing seasons were considered as independent environments differing in climatic conditions, and the objective was to evaluate varietal responses to planting density within each season-specific environment. For each season, data were subjected to analysis of variance with variety, planting density, and their interaction as fixed effects. Where the variety × density interaction was significant, mean comparisons among density levels were conducted separately within each variety to further characterize varietal differences in density response. Before performing analysis of variance and correlation/regression analyses, the data were tested for normality and homogeneity of variance. Linear regression was used to quantify relationships between variables, and the coefficient of determination (*r*^2^) was reported. Graphical representations of data were produced using SigmaPlot 15.0 (Systat Software Inc., Point Richmond, CA, USA).

## 5. Conclusions

This study shows that, under drip fertigation, plant density should be optimized by variety type rather than applied uniformly. Although higher density increased spike number, intercepted radiation, N uptake, and soil water consumption, the two varieties differed in their capacity to convert these gains into grain. MS reached maximum yield at low-to-moderate density (300–400 plants m^−2^) and suffered penalties at the highest density due to reduced post-anthesis RUE, post-anthesis N uptake, NNI (<1.0 at anthesis), harvest index, AE_N_, RE_N_, and WUE, with lodging risk. LS benefited from medium-to-high density (400–500 plants m^−2^), maintaining relatively stable RUE and WUE, higher N status, and sustained N uptake during grain filling, which supported post-anthesis biomass and yield stability. Therefore, the results suggest that, under the conditions of the present study, MS may be better suited to a relatively conservative density strategy, whereas LS may benefit from a relatively intensive density strategy. Further multi-year and multi-location studies are needed to validate these findings and to clarify the physiological and structural mechanisms underlying varietal differences in density response.

## Figures and Tables

**Table 1:** Yield-related agronomic traits of winter wheat in the 2023–2024 and 2024–2025 growing seasons.

Season	Variety	Density	MSN	PSP	SN	GPS	GW	GN	YPS	LAI	LA/G
			(m^–2^)	(%)	(m^–2^)	(Spike^–1^)	(mg)	(10^3^ m^–2^)	(g)		(cm^2^ Grain^–1^)
2023–2024	MS	D2	1104 d	51.5 a	569 c	36.0 a	48.6 a	20.5 b	1.75 a	5.27 c	2.58 c
		D3	1395 c	48.8 b	680 b	34.5 b	46.7 b	23.5 a	1.61 b	6.46 b	2.75 bc
		D4	1639 b	47.2 b	774 a	31.2 c	44.3 c	24.2 a	1.38 c	6.99 a	2.89 b
		D5	1854 a	43.6 c	809 a	26.2 d	42.8 d	21.2 b	1.12 d	7.15 a	3.38 a
		Mean	1498 A	47.8 A	708 A	32.0 B	45.6 B	22.3 A	1.47 B	6.47 A	2.90 B
	LS	D2	832 d	41.3 a	344 c	46.8 a	53.6 a	16.1 c	2.51 a	4.85 c	3.01 a
		D3	1084 c	39.0 ab	422 b	45.0 b	51.3 b	19.0 b	2.31 b	5.96 b	3.13 a
		D4	1297 b	37.1 b	481 a	44.2 b	49.7 c	21.2 a	2.19 b	6.72 a	3.16 a
		D5	1526 a	33.7 c	514 a	42.5 c	47.2 d	21.9 a	2.01 c	6.97 a	3.19 a
		Mean	1185 B	37.8 B	440 B	44.6 A	50.4 A	19.5 B	2.25 A	6.12 B	3.13 A
	ANOVA		*p* value								
	V		<0.001	<0.001	<0.001	<0.001	<0.001	<0.001	<0.001	0.006	<0.001
	D		<0.001	<0.001	<0.001	<0.001	<0.001	<0.001	<0.001	<0.001	<0.001
	V × D		0.182	0.351	<0.001	<0.001	<0.001	<0.001	0.009	0.071	<0.001
2024–2025	MS	D2	1135 d	50.5 a	574 d	35.4 a	44.7 a	20.3 b	1.58 a	5.33 c	2.62 c
		D3	1416 c	48.8 b	691 b	34.1 a	43.0 b	23.6 a	1.47 a	6.52 b	2.77 bc
		D4	1674 b	46.7 c	782 a	30.6 b	41.1 c	24.0 a	1.26 b	7.05 a	2.94 b
		D5	1925 a	42.6 d	819 a	25.4 c	38.1 d	20.8 b	0.97 c	7.20 a	3.46 a
		Mean	1538 A	47.1 A	716 A	31.4 B	41.7 B	22.2 A	1.32 B	6.52 A	2.95 B
	LS	D2	851 d	41.0 a	349 c	47.1 a	49.1 a	16.4 c	2.31 a	5.15 c	3.13 a
		D3	1105 c	38.5 b	426 b	44.6 b	49.0 a	19.0 b	2.19 a	6.00 b	3.16 a
		D4	1321 b	37.1 b	490 a	43.4 b	46.8 b	21.3 a	2.03 b	6.81 a	3.20 a
		D5	1565 a	33.4 c	523 a	41.5 c	45.3 c	21.7 a	1.88 c	7.01 a	3.23 a
		Mean	1221 B	37.5 B	447 B	44.1 A	47.6 A	19.6 B	2.10 A	6.24 B	3.18 A
	ANOVA		*p* value								
	V		<0.001	<0.001	<0.001	<0.001	<0.001	<0.001	<0.001	0.008	<0.001
	D		<0.001	<0.001	<0.001	<0.001	<0.001	<0.001	<0.001	<0.001	<0.001
	V × D		0.167	0.299	<0.001	<0.001	<0.001	<0.001	0.012	0.066	<0.001

Different uppercase letters indicate a significant difference between the means of varieties across four densities according to the least significant difference test (*α* = 0.05). Within a column and a specific variety, different lowercase letters indicate significant differences between plant densities according to the least significant difference test (*α* = 0.05). MSN, the maximum stem number; PSP, productive stem percentage; SN, spike number per m^2^; GPS, grains per spike; GW, grain weight; GN, grain number per m^2^; YPS, yield per spike; LAI, leaf area index; LA/G, the ratio of leaf area to grain number.

**Table 2:** Solar radiation interception and radiation use efficiency of winter wheat in the 2023–2024 and 2024–2025 growing seasons.

Season	Variety	Density	Intercepted Solar Radiation (MJ m^–2^)			Radiation Use Efficiency (g MJ^–1^)		
			Pre-AS	Post-AS	Total	Pre-AS	Post-AS	Seasonal
2023–2024	MS	D2	963.5 d	722.3 b	1685.8 d	1.16 a	1.02 a	1.10 a
		D3	1125.2 c	791.7 a	1916.9 c	1.15 a	1.02 a	1.09 a
		D4	1186.8 b	797.9 a	1984.8 b	1.09 b	0.96 b	1.04 b
		D5	1263.0 a	806.3 a	2069.3 a	1.00 c	0.85 c	0.94 c
		Mean	1134.6 A	779.6 A	1914.2 A	1.10 A	0.96 A	1.04 A
	LS	D2	781.3 c	677.0 c	1458.2 d	1.10 a	0.96 a	1.04 a
		D3	890.8 b	776.9 b	1667.7 c	1.10 a	0.96 a	1.04 a
		D4	1010.2 ab	811.4 ab	1821.6 b	1.10 a	0.96 a	1.04 a
		D5	1047.4 a	830.7 a	1878.1 a	1.09 a	0.92 b	1.02 a
		Mean	932.4 B	774.0 A	1706.4 B	1.10 A	0.95 A	1.03 A
	ANOVA		*p* value					
	V		<0.001	0.386	<0.001	0.071	0.121	0.093
	D		<0.001	<0.001	<0.001	<0.001	<0.001	<0.001
	V × D		0.098	<0.001	<0.001	<0.001	<0.001	<0.001
2024–2025	MS	D2	971.3 d	724.2 b	1695.5 d	1.10 a	0.93 a	1.03 a
		D3	1157.8 c	793.7 a	1951.5 c	1.07 a	0.92 a	1.01 a
		D4	1223.5 b	798.5 a	2022.0 b	1.01 b	0.88 b	0.96 b
		D5	1309.8 a	794.9 a	2104.6 a	0.90 c	0.71 c	0.83 c
		Mean	1165.6 A	777.8 A	1943.4 A	1.02 A	0.86 A	0.96 A
	LS	D2	787.0 d	677.9 c	1464.8 d	1.03 a	0.88 a	0.96 a
		D3	969.4 c	777.2 b	1746.6 c	1.03 a	0.89 a	0.97 a
		D4	1085.5 b	812.0 a	1897.5 b	1.03 a	0.88 a	0.96 a
		D5	1136.7 a	818.8 a	1955.5 a	1.02 a	0.84 b	0.94 a
		Mean	994.6 B	771.5 A	1766.1 B	1.03 A	0.87 A	0.96 A
	ANOVA		*p* value					
	V		<0.001	<0.223	<0.001	0.215	0.097	<0.001
	D		<0.001	<0.001	<0.001	<0.001	<0.001	<0.001
	V × D		0.114	<0.001	<0.001	<0.001	<0.001	<0.001

Different uppercase letters indicate a significant difference between the means of varieties across four densities according to the least significant difference test (*α* = 0.05). Within a column and a specific variety, different lowercase letters indicate significant differences between plant densities according to the least significant difference test (*α* = 0.05). Pre-AS, pre-anthesis; post-AS, post-anthesis.

## Data Availability

All data supporting this study are included in the article.
